# Chronic Stress and Cardiovascular Disease among Individuals Exposed to Lead: A Pilot Study

**DOI:** 10.3390/diseases8010007

**Published:** 2020-03-15

**Authors:** Emmanuel Obeng-Gyasi, Barnabas Obeng-Gyasi

**Affiliations:** 1 Department of Built Environment, North Carolina Agricultural and Technical State University, Greensboro, NC 27411, USA; 2 Department of Biological Sciences, Purdue University, West Lafayette, IN 47907 USA; bobenggy@purdue.edu

**Keywords:** lead exposure, chronic stress, lead cardiovascular, allostatic load

## Abstract

Chronic stress and cardiovascular disease risk were explored in a predominately middle-aged adult population exposed to elevated lead levels in this cross-sectional study using data from the National Health and Nutrition Examination Survey (NHANES) from the period 2007-2010. Elevated lead exposure was defined using the epidemiological threshold of a blood lead level (BLL) > 5 μg/dL as defined by the U.S. Centers for Disease Control and Prevention (CDC). Allostatic load (AL), a measure of chronic stress, was operationalized using 10 clinical markers. The geometric mean values for clinical cardiovascular disease risk markers of interest (a) Gamma glutamyl-transferase (GGT) (a marker of oxidative stress), and (b) non-HDL cholesterol (non-HDL-c) (a marker of cardiovascular disease risk) were explored among lead-exposed and less lead-exposed individuals with differential chronic stress (AL) levels. Associations between AL and GGT/non-HDL-C were analyzed using linear regression models. The likelihood of increased clinical markers in lead-exposed individuals with high compared to low AL was explored using binary logistic regression models. In analyzing lead-exposed as compared to less lead-exposed populations, the geometric mean of the variables of interest showed significant elevations among lead-exposed individuals as compared to less lead-exposed individuals. Simple linear regression revealed that AL was positively associated with the variables of interest among the lead-exposed. In binary logistic regression among the lead-exposed, those with high AL, as compared to those with low AL, had significantly higher odds of having elevated non-HDL-C. This study submits that those exposed to lead with increasing AL may experience adverse cardiovascular health outcomes.

## 1. Introduction

Lead exposure arises from many sources in different environments [1,2]. It begins as early as pregnancy [3], affects almost every physiological system within the human body [4,5,6,7,8,9], and harms those exposed throughout their life course [10,11]. Factors such as socio-economic and environmental stress [12], when combined with lead exposure over a prolonged period, may initiate or promote disease [13]. Allostatic load (AL), a critical marker of chronic physiological stress, represents dysregulation across many physiological systems brought forth in response to persistent environmental burdens and reflects the cumulative biological burden exacted by this demand on the body [14,15,16,17]. AL represents the hypothalamic–pituitary–adrenal axis in an over-activated state and involves the dysregulation of multiple physiological systems [18]. This is because the body’s response to stress causes the neuroendocrine system to be activated, and mediators such as cortisol, epinephrine, and norepinephrine are released, bringing forth a cascade of effects on systems such as the cardiovascular, metabolic and immune systems. Recurrent, lengthy, or insufficient stress responses induce systemic dysregulation in multiple systems and can ultimately lead to diseases [19]. AL ultimately represents a process from stress to diseases and provides a means for understanding the damaging effects of recurrent or chronic stress on adverse health outcomes.

In the U.S. population, cardiovascular diseases are responsible for the highest percentage of mortality [20]. Markers of cardiovascular health, such as blood pressure (SBP and DBP), are commonly used to evaluate the state of the cardiovascular system. Numerous epidemiological studies have linked lead exposure with hypertension [21,22], and with increases in blood lead level (BLL) increasing blood pressure [8,23,24,25].

Non-HDL cholesterol (non-HDL-C), when compared to LDL cholesterol, serves as a superior marker for cardiovascular disease risk [26] with elevated levels indicating worse outcomes [27,28]. Gamma-glutamyl transferase (GGT), which is present in several cell types, can be used as a marker of oxidative stress [29,30]—a state in which pro-oxidants overwhelm antioxidants and free reactive oxygen species (ROS)—and induce damage to physiological systems [31].

**Figure 1:**
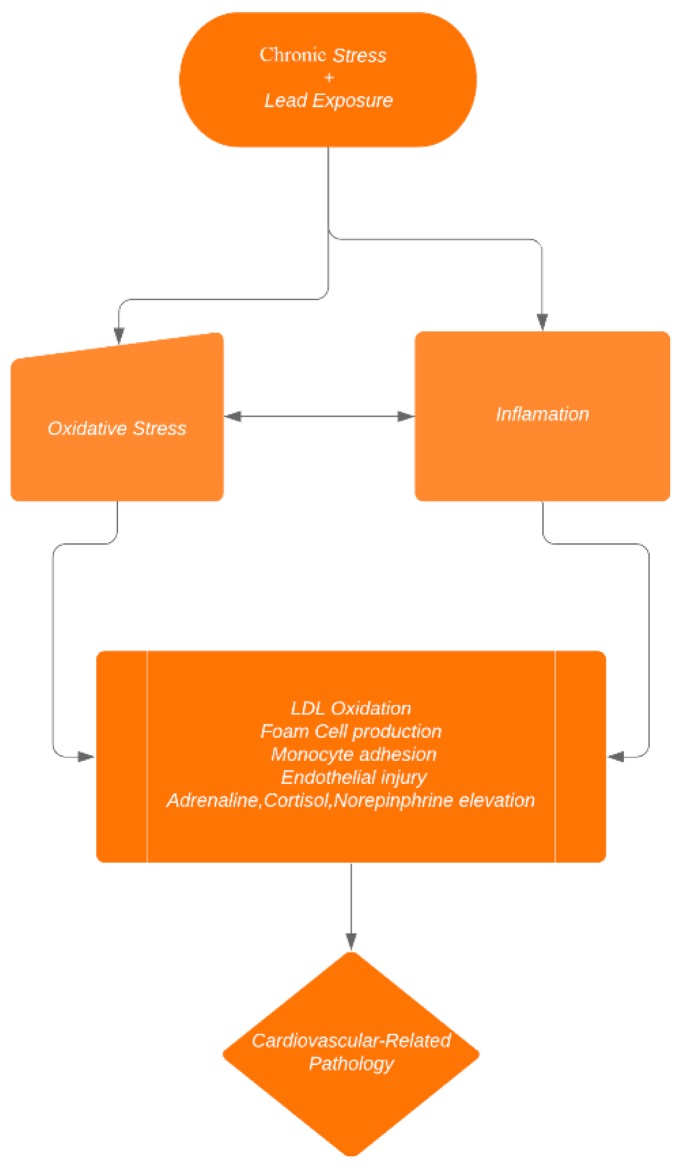
Mechanisms of stress-induced cardiovascular dysfunction among the lead-exposed.

Epidemiological studies have demonstrated that acute and chronic stress predicts the incidence of cardiovascular dysfunction. Indeed, individuals who experience work-related stress have an increased risk of cardiovascular health issues, since chronic stress at the workplace and in private life is associated with various cardiovascular diseases [32]. Alterations in autonomic along with hemostatic and inflammatory processes are critical mechanisms by which physiological stress triggers cardiovascular dysfunction [32]. The potential mechanism by which stress and lead alter cardiovascular dysfunction is found in Figure 1 below.

According to the Centers for Disease Control and Prevention (CDC), all exposure to lead can induce pathology, with exposure levels greater than the 5 μg/dL threshold considered to be elevated for children and adults [33]. The health outcomes and physiological associations of lead exposure, and the risk for many significant conditions, are altered by multiple physiological systems such as the cardiovascular, inflammatory and metabolic systems. This indicates that AL indices that use multiple systems provide the best picture of the physiological burden that lead exposure has on the human body. The relationship between chronic stress, and markers of cardiovascular disease (non-HDL-C and GGT) are examined in this study among individuals with elevated lead levels in order to determine the role that stress plays on these markers.

## 2. Materials and Methods

### 2.1. Hypothesis

The hypothesis of this study is that, among those exposed to elevated lead levels, chronic physiological stress, as measured by AL, increases oxidative stress, and heart disease risk. The objectives of this study were therefore to examine the effects of AL on non-HDL-C and GGT in lead-exposed individuals, as defined by the CDC threshold of (BLL) > 5 μg/dL.

### 2.2. Research Design

Variables were chosen based on their availability in the National Health and Nutrition Examination Survey (NHANES) dataset. The relationship between chronic stress (AL), heart disease risk (non-HDL-C) and oxidative stress (GGT) was explored using NHANES 2007-2010. NHANES data are a stratified, multistage probability sample of civilian non-institutionalized individuals in all of the fifty U.S. states, including the District of Columbia. The technical details of the survey, including sampling design, data collection protocols, and data availability, are freely available on their website.

#### 2.2.1. Operationalising Allostatic Load

Informed by prior studies [34], AL was operationalized by developing a cumulative index of physiologic dysfunction of the cardiovascular (SBP, DBP, triglycerides, HDL cholesterol, total cholesterol), inflammatory (CRP), and the metabolic systems (BMI, hemoglobin A1C, albumin, creatinine clearance). AL biomarkers were divided into quartiles based on their distribution within the database. High-risk for each biomarker was considered to be the top 25% in the distribution for all markers apart from albumin, creatinine clearance, and HDL cholesterol, for which the bottom 25% of the distribution was considered to show the highest risk, as determined by the literature [8,35,36,37,38,39,40]. Each individual in the study was assigned a value of 1 if they were in the high-risk category or a 0 if in the low-risk category for all markers to calculate a total AL value out of 10. The data were then analyzed examining the relationship between AL and the clinical markers of interest.

#### 2.2.2. Data collection in NHANES

During the in-home interview, a standardized questionnaire was used to collect demographic information including age, race–ethnicity, and sex. During participants’ visit to the mobile examination center (MEC), height and weight were measured and body mass index was calculated. A blood specimen was drawn from the participant’s antecubital vein by a trained phlebotomist according to a standardized protocol. Whole blood samples served as the medium to analyze lead in NHANES 2007-2010 via inductively coupled plasma mass spectrometry (ICP-MS). An aliquot of urine was shipped to the University of Minnesota for urinary creatinine and albumin analysis. Creatinine was measured using a Jaffe rate reaction with a Beckman Synchron CX3 clinical analyzer (Beckman Coulter, Fullerton, CA, USA). Urine albumin was measured using a solid-phase fluorescent immunoassay, and fluorescence was determined with a Sequoia–Turner Digital fluorometer (Sequoia–Turner Corp., Mountain View, CA, USA). A1C was measured using a Tosoh A1C 2.2 Plus Glycohemoglobin Analyzer or a Tosoh G7 Automated HPLC Analyzer (Tosoh Medics, Inc, San Francisco, CA, USA). CRP was measured using latex-enhanced nephelometry on a Behring Nephelometer Analyzer System (Behring Diagnostics, Inc, San Jose, CA, USA) with NA Latex CRP Kit (Behring Diagnostics, Inc, San Jose, CA, USA). Fasting total serum cholesterol, along with triglycerides, were measured enzymatically on a Roche/Hitachi Modular P Chemistry Analyzer (Roche Diagnostics Corp, Indianapolis, IN, USA). HDL cholesterol was measured using a modification of the traditional multistep precipitation reaction.

Biochemistry biomarkers were measured using a Beckman Synchron LX20 and Beckman Coulter UniCel^®^ DxC800 (Beckman Coulter, Fullerton, CA, USA). Stata SE/16.0 (StataCorp, College Station, TX, USA) performed the data analysis, as this allowed for the adjustment needed to account for the complex design.

### 2.3. Data Analysis

The data in this cross-sectional study were analyzed for lead-exposed individuals (those with BLLs above and below 5 μg/dL) for the effects of stress on cardiovascular-related markers (non-HDL-C and GGT).

The geometric mean values of the markers were firstly examined in those exposed above the CDC-defined 5 μg/dL exposure level (known in this study as the lead-exposed) and those below the 5 μg/dL exposure level (known in this study as the less lead-exposed) to determine the baseline differences in the population.

Simple linear regression was performed to examine the associations between AL and the markers of interest (non-HDL-C and GGT) among lead-exposed individuals and less lead-exposed individuals above and below the BLL-5 μg/dL threshold. In addition, these linear regression models were examined at the BLL-3 μg/dL threshold level in order to have a larger sample size for the exposed population and have more robust regression models. The 3 μg/dL threshold has been demonstrated to cause worse health outcomes in several studies [41,42,43]. The data were adjusted for age, gender, BMI, ethnicity, alcohol consumption, and smoking based on the literature [44,45,46,47,48].

The odds of elevated chronic stress, as defined by an AL binary at 4 for high versus low levels, was explored in the lead-exposed individuals via age-adjusted binary logistic regression models, as having an AL above this level has been consistently shown in the literature to be a high risk [49,50,51]. Each exposure–outcome combination was examined in individual models.

In this study, when exploring cardiovascular makers among lead-exposed individuals, AL was the dependent variable, with the clinical markers of interest (non-HDL-C- and GGT) being the independent variables. Stata SE 16.0 was factored in the complex design to ensure the analysis reflected the proper weights and was guided by the tutorial [52] provided by the NHANES. The Shapiro–Wilk test revealed that all of the variables lacked a normal distribution, so they were natural log-transformed. P-values less than or equal to 0.05 were considered significant.

## 3. Results

### 3.1. Study Variables Among Lead-Exposed and Less Lead-Exposed Participant

The geometric mean values of lead-exposed (≥5 μg/dL) vs. less lead-exposed (≤5 μg/dL) individuals were analyzed for all critical variables in this study. The values for lead-exposed individuals were generally more elevated as compared to less lead-exposed individuals. The results can be found in Table 1 below.

### 3.2. Geometric Mean GGT and Non-HDL-C in Lead-Exposed Individuals with High/Low Allostatic Load

The geometric mean of the clincal variables of interest among lead-exposed (BLL > 5 µg/dL) and less lead-exposed indivials (BLL < 5 µg/dL) with high AL (greater than 4) and low AL (less than 4) were explored. The results can be found in Table 2 below.

### 3.3. Likelihood of Elevated Clinical Markers at AL Binary at 4 in Individuals Exposed to Lead

Age-adjusted binary logistic regression models were used to predict the odds of elevated clinical markers. The binary dependent variable was AL (high/low) at the level of 4. This was done among those exposed to lead and was analyzed in 246 individuals. A positive significant association was found between lead-exposed individuals and non-HDL-C. The results were that that those with an elevated allostatic load were more likely to have a higher cardiovascular disease risk, as represented by non-HDL-c (OR 1.01; 95% CI 0.99–1.03; *p* = 0.001). GGT showed a similar trend but was not statistically significant.

### 3.4. Association of AL with Markers of Interest in Lead-Exposed Individuals

The relationship between AL and the markers among lead-exposed (LE) at the 5 µg/dL level and less lead-exposed (LLE) individuals was explored using linear regression. Among those exposed to lead, there was a more positive but non-significant association between AL and the markers of interest. The results are found in Table 3.

A similar analysis was performed, with lead-exposed being those with BLL above 3 µg/dL for lead-exposed, as the sample size was smaller in the 5 µg/dL group. Increasing the sample size of the exposed resulted in non-HDL-C becoming statistically significant with a similar magnitude. The results are shown in Table 4.

## 4. Discussion

Allostatic load is a multiple-system biomarker of the biological burden brought forth by the ongoing disruption of the body’s response to chronic stress [53]. This study sought to examine the associations between AL and clinical cardiovascular makers in lead-exposed individuals and less lead-exposed individuals. It discovered that, in lead-exposed individuals, AL was more elevated in Blacks as compared to Whites, and in males as compared to females. This bolsters the work of Geronimus and colleagues, which found that Blacks exhibited a higher allostatic load than Whites [49]. This study finding is also in agreement with the weathering hypothesis [54], which suggests that the health of Blacks prematurely worsens owing to racial disparities [14,55]. This study’s finding of higher stress levels when combined with lead hints that the most at-risk populations may bear the worst outcome of cumulative exposure. Previous studies have noted associations between general distress and blood lead levels (BLLs) [56] and bone lead levels with phobic anxiety [57], which further suggests that these things tend to occur together in many environments.

This study is one of the first of its kind to look at AL among lead-exposed individuals, examining markers of cardiovascular disease risk (non-HDL-C) and oxidative stress (GGT). It discovered positive associations between AL cardiovascular disease risk among lead-exposed individuals, demonstrating that lead-exposed individuals exposed to increasing stress are significantly more likely to have elevated adverse cardiovascular outcomes. In regression models in this study, the coefficient was more positive in the lead exposed group than the less-lead exposed group, potentially indicating that the added effect of lead worsens outcomes. In addition, the geometric mean levels of the markers of interest were more elevated among lead-exposed individuals and those with a higher allostatic load. The above-mentioned findings bolster an earlier study by Peters et al., which found a significant relationship between bone lead levels, a measure of long term exposure, and stress among those with hypertension in a study in Boston, U.S.A [13].

The association of AL with markers of cardiovascular disease-risk in lead-exposed individuals is likely due to the release of hormones such as epinephrine, dehydroepiandrosterone sulfate, and cortisol, which play a role in cardiovascular disease-risk as measured by variables such as non-HDL-C [58].

Though oxidative stress was elevated in the lead-exposed individuals, it was not found to be a significant player in AL among those exposed at the 5 µg/dL level; the AL model suggests that recurrent alterations in homeostasis are connected to oxidative stress and inflammatory responses. The trends of this study did demonstrate that associations were positive but not significant, which may suggest that a larger sample size or different markers for oxidative stress may better capture this. This is bolstered by the results at the 3 µg/dL level, which had a larger sample size and demonstrated significantly worse outcomes.

This study critically adjusted for factors such as smoking and alcohol consumption. Cigarettes may contain traces of lead, and some alcohol, depending on how it was brewed, may contain lead. In addition, stress may cause individuals to engage in behaviors which increase the consumption of both.

Ultimately, behaviors such as physical exercise, in addition to the availability of social support to increase resilient behaviors, can help to manage AL. Lower stress can be best achieved by lowering AL and increasing resilience, as the long-term consequence of chronic, unrelieved, multi-year stress is ultimately death as it produces pathological changes and exacerbates life-style diseases such as adult-onset diabetes and atherosclerosis.

The limitations of this study include the fact that the study is cross-sectional, and therefore does not allow for any deduction of causality or even temporal relationships. The study also did not include institutionalized persons, such as individuals in nursing homes or those in prisons. This means that the dataset inherently has some selection bias. Future works should seek to collect samples among urban and peri-urban minorities and hard-to-reach populations who are exposed to higher stress and lead levels to determine the unique challenges of such exposure among these critical and vulnerable populations.

## 5. Conclusions

Cardiovascular diseases are multifactorial, with physical and social environments playing a significant role in their pathogenesis. Among the individuals differentially exposed to lead, AL adversely affects cardiovascular outcomes in those with stress. Public health practitioners must work to mitigate both physical and social exposures to lead and promote resilient behaviors to limit the cumulative effects of lead and stress on human health.

## Figures and Tables

**Table 1:** The geometric mean values of lead-exposed vs. less lead-exposed of all critical variables in this study.

*Variable*	*Lead-Exposed* (BLL ≥ 5 µg/dL)	*Less Lead-Exposed* (BLL < 5 µg/dL)	*p-Value*
N	255	9526	
Mean Age (95% CI)	49.04 (46.95–51.15)	38.27 (37.70–39.05)	<0.001
Allostatic Load by Ethnicity (95% CI)			
Non-Hispanic Black	2.94 (2.66–3.23)	2.24 (2.14–2.34)	<0.001
Non-Hispanic White	2.67 (2.42–2.92)	2.43 (2.35–2.52)	0.046
Allostatic Load by Gender (95% CI)			
Male	2.68 (2.42–2.93)	2.39 (2.33–2.46)	0.032
Female	2.31 (1.64–2.98)	2.35 (2.27–2.44)	0.885
Non-HDL-C (95% CI)	152.12 (143.36–160.88)	139.31 (138.08–140.54)	0.006
GGT (95% CI)	39.34 (31.22–47.46)	26.12 (25.24–27.01)	<0.001
Smoking every day percent (95% CI)	52.09 (45.79–58.33)	38.88 (35.89–41.96)	0.001
Alcohol percent (95% CI)	84.96 (77.90–90.05)	75.81 (73.57–77.93)	0.007

**Table 2:** The geometric mean values of lead-exposed vs. less lead-exposed among those with high and low AL for clinical variables of interest.

*Variable*	*Lead-Exposed* (BLL ≥ 5 µg/dL)	*Less Lead-Exposed* (BLL < 5 µg/dL)
Non-HDL-C (95% CI) (High AL)	167.48 (155.53–179.43)	163.71 (161.78–164.64)
Non-HDL-C (95% CI) (Low AL)	144. 77 (135.34–154.20)	131.82 (130.63–133.02)
GGT (95% CI) (High AL)	49.51 (30.73–68.30)	33.33 (31.36–35.31)
GGT (95% CI) (Low AL)	34.77 (27.12–42.43)	23.92 (23.02–24.84)

**Table 3:** Simple linear regression—relationship of AL with cardiovascular-markers in lead-exposed (LE) individuals and less lead-exposed (LLE) individuals at the 5 µg/dL threshold.

*Variables*	*lnAL Adjusted (95% CI) +*	*p-Value*
GGT (LE)	0.30 (−0.12, 0.72)	0.148
nonHDL-C (LE)	0.79 (−0.13–1.70)	0.088
GGT (LLE)	0.10 (0.03, 0.17)	0.009
nonHDL-C (LLE)	0.60 (0.50–0.71)	<0.001

+ adjusted for age, BMI, gender, alcohol consumption, smoking, in addition to taking prescription medicines for hypertension. LE = Lead-Exposed, LLE = Less Lead-Exposed.

**Table 4:** Simple linear regression—relationship of AL with cardiovascular-markers in lead-exposed (LE) and less lead-exposed (LLE) individuals at the 3 µg/dL threshold.

*Variables*	*lnAL Adjusted (95% CI) +*	*p-Value*
GGT (LE)	0.13 (0.01, 0.25)	0.036
nonHDL-C (LE)	0.73 (0.48–0.97)	<0.001
GGT (LLE)	0.10 (0.02, 0.18)	0.013
nonHDL-C (LLE)	0.596 (0.48–0.71)	<0.001

+ adjusted for age, BMI, gender, alcohol consumption, smoking, in addition to taking prescription medicines for hypertension. LE = Lead-Exposed, LLE = Less Lead-Exposed.
